# G-Quadruplex Unwinding Molecular Mechanisms by Helicases and Their Applications

**DOI:** 10.3390/ijms27041629

**Published:** 2026-02-07

**Authors:** Jiawen Sun, Yangzhi Wang, Yihua Huang, Zhongzhou Chen

**Affiliations:** 1 State Key Laboratory of Animal Biotech Breeding, College of Biological Sciences, China Agricultural University, Beijing 100193, China; sz20243020415@cau.edu.cn; 2 State Key Laboratory of Biomacromolecules, Institute of Biophysics, Chinese Academy of Sciences, Beijing 100101, China; wangyangzhi21@mails.ucas.ac.cn; 3 University of Chinese Academy of Sciences, Beijing 100101, China

**Keywords:** G-quadruplex, helicase, unwinding, structural basis, telomere, drug design, G4 ligand, nanopore sequencing

## Abstract

G-quadruplexes (G4s) are specialized nucleic acid structures extensively formed throughout the genome, with particular enrichment in regulatory regions such as telomeres, promoters, and transcriptional enhancers. These four-stranded assemblies are involved in multiple chromosomal processes, including DNA replication, transcription, maintenance of genomic stability, and epigenetic regulation, and are closely associated with cancer biology. Due to their unusual thermodynamic stability, G4s serve as physical barriers to DNA/RNA unwinding, thereby impeding replication, transcription, and translation and compromising genome integrity. To mitigate this threat, cells have evolved dedicated helicases that can actively resolve G4 structures. In this review, we summarize recent structural advances—primarily derived from protein crystallography—regarding the mechanisms by which helicases unwind G4 quadruplexes. The insights presented herein establish a framework for elucidating the molecular basis of G4 unfolding and for the rational design of small-molecule G4 ligands and therapeutic agents. Additionally, we explore the applications of G4 helicases in nanopore sequencing, which aim to enhance sequencing accuracy, throughput, and continuity.

## 1. Introduction

**Figure 1:**
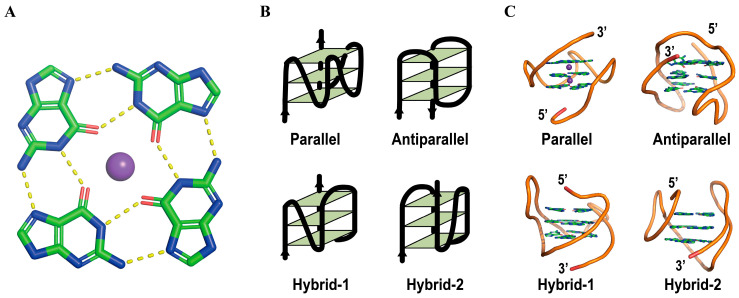
Schematic representation of G4s. (**A**) The planar G-tetrad, stabilized by Hoogsteen hydrogen bonds and a centrally coordinated metal ion (typically K^+^, purple). Carbon, green; nitrogen, blue; oxygen, red. (**B**) Successive tetrads can stack in diverse relative orientations to form the four-stranded architecture [11]. (**C**) Schematic representation of the diverse conformations of the G4 quadruplex. Parallel PDB: 1XAV, Antiparallel PDB: 143D, hybrid PDB: 2JSL, PDB: 2JSM.

G-quadruplexes (G4s) are atypical DNA/RNA secondary structures formed by guanine-rich sequences [1,2]. Each G-tetrad comprises four guanine bases linked via hydrogen bonds involving Hoogsteen base pairing [3,4], and stabilized by cations (such as K^+^ and Na^+^) in the central channel of the G4 helix (Figure 1A). Vertical stacking of multiple G-tetrads yields a highly stable four-stranded architecture [5,6,7]. In the context of human double-stranded genomic DNA, G4 structures can persist in a stabilized form. However, in the presence of complementary DNA, their inherent thermodynamic stability is lower than that of the corresponding Watson–Crick duplex [8]. The observed stability is therefore conditional, being dictated by topological constraints, protection afforded by protein or ligand binding, and the chromatin dynamics associated with cell-cycle progression [8,9,10]. G4s can be classified into three categories: antiparallel, parallel, and hybrid topologies (Figure 1B,C) [11]. About 10,000 G4 structures were detected in human chromatin, primarily within regulatory regions and nucleosome-depleted regions [12,13,14,15]. G4s are enriched in the promoter regions of various genes, particularly oncogenes and loci implicated in cellular responses to external stimuli, growth regulation, intercellular signaling, and cell motility [16,17,18,19,20,21]. For example, telomeres, consisting of thousands of the consensus sequence TTAGGG repeats in mammals, protect the chromosome from shortening or degradation during replication [22,23].

In genomic components presumed to be functional, G4s are significantly over-represented, possess high predicted thermostability, and evolve under detectable purifying selection. These characteristic G4s are most evident in CpG islands, promoters and both 3′ and 5′ untranslated regions (UTRs). In contrast, G4 motifs located on the non-transcribed strand of protein-coding exons are under-represented, structurally unstable, and evolutionarily neutral. Consistently, G4 density and stability are lower on the non-transcribed strand than on the transcribed strand across all genic features [24,25]. The presence of G4 structures impedes DNA/RNA unwinding and replication, leading to stalling of the replication fork, microsatellite instability, mutagenesis, chromosomal rearrangements, and translation [26]. Current studies have shown that the *Escherichia coli* genome harbors a large number of potential G4 motifs, which can fold into stable structures under specific conditions (e.g., elevated K^+^ concentrations) [27,28,29]. In vitro experiments have demonstrated that certain G4 formations impede DNA polymerase I (Pol I)—mediated replication, suggesting that they may interfere with DNA transactions linked to transcription or replication. To overcome the adverse impact, cells have evolved multiple helicases capable of resolving G4 structures, including the superfamily 1 (SF1) helicase Pif1, several SF2 helicases such as DHX36, XPD-family members (FANCJ and RTEL1), and RecQ-family helicases (BLM, WRN, and RECQ1) [13,30,31]. Structural elucidation of these helicases and mechanistic dissection of their unwinding activity have provided new avenues for rational drug design.

## 2. Helicases

### 2.1. DHX36 Helicases

**Figure 2:**
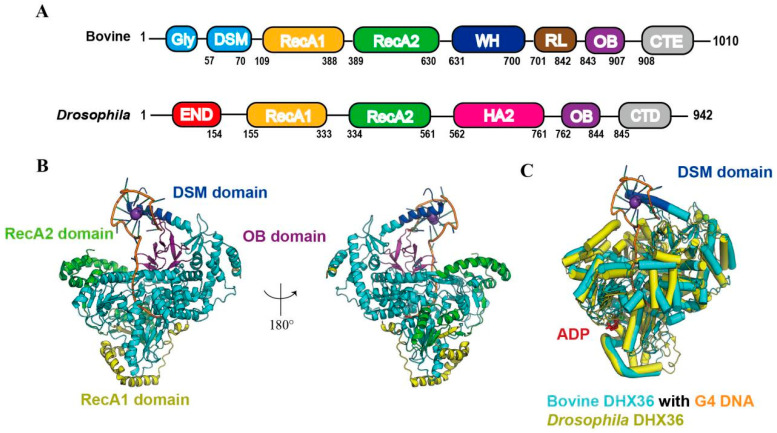
Structural schematic of DHX36 [40]. (**A**) The domain organization of bovine and *Drosophila* DHX36. (**B**) The DSM domain, in concert with the RecA domain, engages the G4 and propels the helicase along the nucleic-acid track. 180° rotation shown in the right panel. (**C**) Structural superposition of bovine DHX36 (PDB code: 5VHE, cyan) and *Drosophila melanogaster* DHX36 (PDB code: 5AOR, yellow).

DHX36, also designated RHAU or G4R1, is a member of the DEAH/RHA helicase family that exhibits high-affinity binding to both DNA and RNA G4s [12,32,33]. Human DHX36 is functionally linked to cardiac development, hematopoiesis, and telomere maintenance [34,35]. Additionally, other DEAH-family members, such as RTEL1 and eIF4A, have also been demonstrated to possess G4-unwinding activity [36]. DHX36 directly binds to and unwinds the G4 DNA on the chromatin, preventing the occurrence of DNA double-strand breaks during replication and transcription, thereby maintaining genomic integrity and supporting normal cell proliferation [37]. Structural analyses have identified an N-terminal DHX36-specific motif (DSM) module (Figure 2A) that is indispensable for G4 recognition [34,38], and subsequent high-resolution structures have laid the groundwork for elucidating the molecular basis of G4 unwinding [39,40].

Using X-ray crystallography, Chen et al. determined the crystal structure of the bovine DHX36 in complex with the c-Myc G4, revealing that DNA binding induces the DSM to fold into two α-helices whose hydrophobic residues (Y69/W68/I65) form π-π stacking or hydrophobic interactions with the top G-tetrad [40]. The oligonucleotide/oligosaccharide-binding (OB) domain concurrently engages the 3′-single-stranded tail [39,40] (Figure 2B). The two RecA-like domains of bovine DHX36 (RecA1 and RecA2) form a positively charged channel that clamps the 3′-single-stranded DNA tail emerging from the G4. A network of polar residues on the RecA2 surface stabilizes the G4 phosphate backbone. Subsequent ATP-driven rotation of RecA2 initiates local G4 collapse and strand pulling, thereby coupling ATP hydrolysis to directional G4 unwinding. Upon complex formation, the uppermost G-tetrad is pulled apart, with nucleotide G17 being displaced into the single-stranded region. The remaining three layers are stabilized by a non-canonical quartet, leading to an overall reduction in G4 stability and generating the tension required for stepwise strand separation. DHX36-mediated repetitive unfolding of G4s is ATP-independent. ATP is required only for the final step of enzyme dissociation from DNA. This study provided the first demonstration that a DEAH helicase can harness nucleic acid-binding energy to drive domain rotation, offering a new framework for transcriptional regulation and anticancer drug design [40].

Complementary structural work on the *Drosophila melanogaster* ortholog (DmDHX36) in complex with RNA and a series of DNA substrates showed that DmDHX36 retains the DSM-OB-RecA2 architecture characteristic of mammalian DHX36 with a five-amino-acid deletion in its DSM. The N-terminal region is dispensable for G4 binding. In contrast, the human DSM motif is essential [41]. Superposition of the RecA domains of bovine DHX36 (PDB: 5VHE) and *Drosophila melanogaster* DHX36 (PDB: 5AOR) reveals an almost identical motor scaffold (Figure 2C). However, bovine DHX36 carries an N-terminal DSM-α1 helix that docks onto the outer face of the top G-tetrad, forming π–π and cation–π contacts with the guanine bases; this stacking interaction underlies its exclusive specificity for parallel G4 structures [40]. Both orthologs employ a positively charged surface to locally destabilize the G4, which is then driven to complete unwinding by ATP-dependent translocation [42]. Single-molecule assays further revealed that DHX36 operates in two distinct modes—G4 protection and G4 unfolding—with the switch governed by ATP-hydrolysis-driven conformational rearrangements rather than ATP binding perse [43]. When DHX36 binds the G4 structure solely via its DSM domain, it exerts a protective effect without unwinding the G4. Upon ATP hydrolysis, conformational changes in the RecA domain locally disrupt the G4. Once unwinding is completed, the helicase returns to its initial state and enters a new cycle, thereby achieving multiple rounds of G4 unfolding. The DSM and the 3′-single-stranded tail act synergistically, thereby providing a druggable interface for modulating G4 metabolism.

DHX36 evolves a movable RecA2 insertion loop within the conserved DEAH-box core, working in conjunction with the N-terminal DSM/OB module [44]. This enables it not only to recognize the common planar features of G4 structures, but also to distinguish the sugar-phosphate differences between DNA and RNA. Thus, it achieves efficient unwinding of both types of G4 structures within the same structural framework. DHX36 converts ATP-binding energy into a directed, single-base stepping force, accounting for its strict preference for parallel G4s and its exceptionally high unwinding efficiency. This mechanism furnishes a novel protein–nucleic-acid interface template for the design of G4-ligand antagonists or G4-targeted antineoplastic agents [42].

### 2.2. Pif1 Helicases

Pif1 helicase belongs to the SF1B superfamily and translocates in the 5′→3′ direction along DNA. In vivo, it acts directly on G4 DNAs and efficiently unwinds them, thereby preventing DNA damage, chromosomal rearrangements and epigenetic aberrations—a function that is highly conserved from prokaryotes to eukaryotes [45,46]. Members of the Pif1 family exhibit a strong preference for G4 substrates, and the presence of G4 structures markedly stimulates their helicase activity [47,48].

The crystal structure of the thermophilic bacterium “*Thermus oshimai*” Pif1 (ToPif1) in complex with parallel-stranded G4 DNA reveals that its 1B and 2B sub-domains constitute a dedicated G4- Recognizing Surface (GRS) that docks onto the ribose-phosphate backbone of the G4 [49]. The binding of G4 to ToPif1 is mediated primarily by electrostatic contacts, hydrogen bonds and ionic interactions, but without base-specific recognition. In contrast to the ATP-independent, repetitive unfolding mechanism observed for SF2 helicases such as bovine DHX36, ToPif1 unwinds G4 structures in an ATP-dependent, stepwise and essentially irreversible manner.

**Figure 3:**
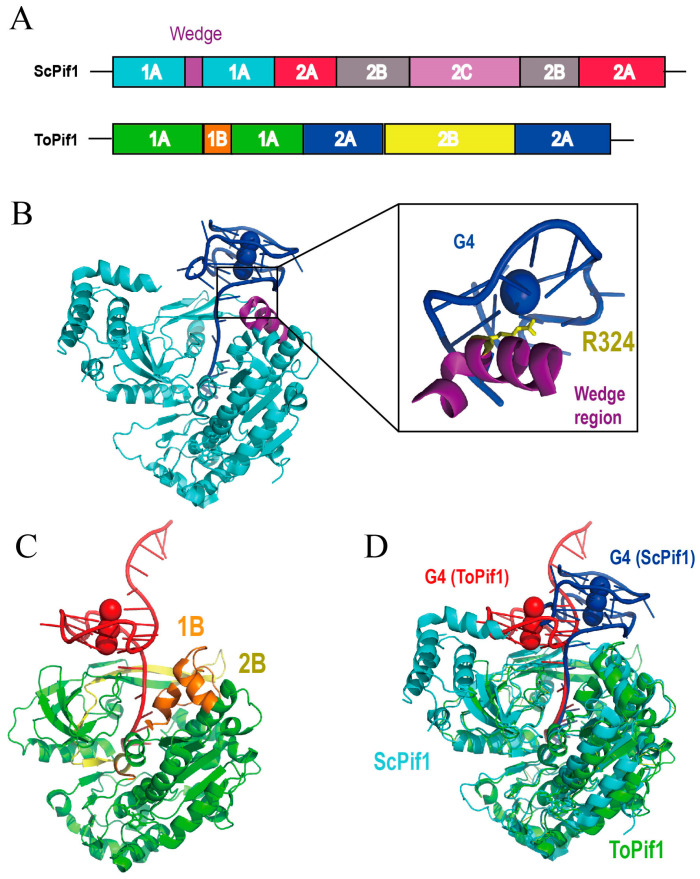
Schematic representation of the architecture of Pif1. (**A**) The domain organization of bovine and *Drosophila* DHX36. (**B**) Crystal structure of ScPif1 (PDB code:8XAK, cyan), illustrating the wedge region (purple) within the 1A domain that recognizes and binds the G4 (blue) [50]. The key residue R324 involved in the interaction is shown as yellow sticks. (**C**) Crystal structure of ToPif1 (PDB code: 7OAR, green), showing the 1B/2B domains forming the G4-recognition platform [49]. (**D**) Structure alignment of ToPif1 and ScPif1. The parallel G4 is rotated approximately 180° as a whole, with the 5′-tetrad oriented in the opposite direction.

*Saccharomyces cerevisiae* Pif1 exhibits exceptional proficiency in unwinding G4 structures and facilitates DNA polymerase, including Pol δ, POL γ and Mip1, to traverse G4-containing templates [1,30]. ScPif1 anchors the first 5′-terminal G-tetrad via a wedge region (such as key residue R324) located within the 1A domain, while its oligonucleotide/oligosaccharide-binding (OB) fold engages the 3′ single-stranded tail (Figure 3B). ATP hydrolysis drives stepwise unstacking, with the first guanine base G9 in the G4 being translocated, yielding a G3-triplex intermediate [50]. This sequential mechanism appears conserved among eukaryotic Pif1 orthologs. The same wedge region also recognizes the ssDNA-G4 junction, furnishing an entry point for G4 unfolding and enabling a unified mode of substrate engagement. By preferentially eliminating G4 barriers ahead of the replication fork, ScPif1 suppresses genome instability and chromosomal rearrangements.

In contrast to ToPif1, the 2B domain of ScPif1 merely contacts peripheral regions of the G4 and is not involved in core recognition. The wedge region of ToPif1 makes no direct contact with the G4 during G4 unwinding and is therefore dispensable for the unfolding reaction of this substrate (Figure 3C). The G4 orientation in the ScPif1 complex is rotated approximately 180° relative to that in the ToPif1 complex, underscoring significant inter-species variations in G4 recognition mechanisms within the Pif1 family (Figure 3D). Comparative analysis of the RecA1 domain across Pif1 orthologs from diverse species reveals a conserved motor protein core, with species-specific variations confined to the wedge region.

### 2.3. XPD Helicases

XPD helicase is one of the 5′→3′ SF2 superfamily that shares homology with established G4 resolvases such as FANCJ, RTEL1 and CHL1, and constitutes an essential component of nucleotide-excision repair (NER) [51]. G4-unwinding activity has been documented only in select orthologues (e.g., *Sulfolobus acidocaldiarius* XPD (SaXPD)) and does not represent a universal property of the family. For instance, *Thermoplasma acidophilum* XPD (TaXPD) lacks demonstrable G4 resolvase activity [52]. In humans, XPD is a core subunit of the transcription-repair factor TFIIH, and any G4-related function is likely structural or indirect. Nevertheless, its biological significance remains evident, particularly in the unwinding of promoter-associated G4 structures.

**Figure 4:**
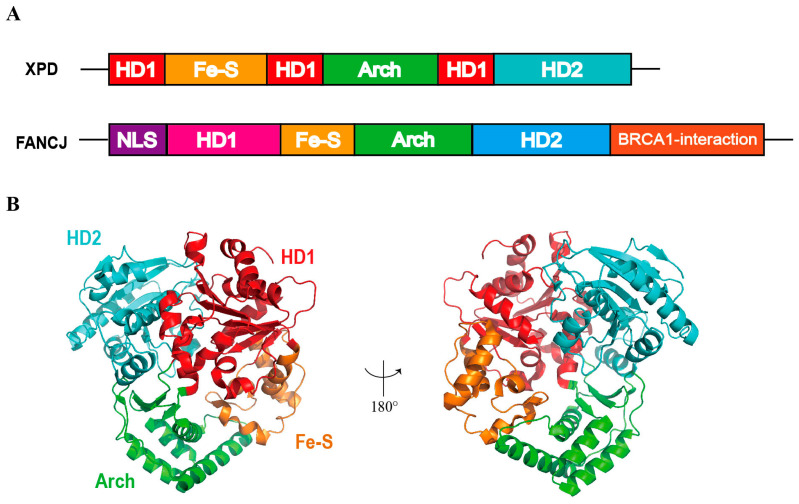
Schematic representation of the architecture of XPD. (**A**) The domain organization of XPD and FANCJ. (**B**) Crystal structure of SaXPD (PDB code: 3CRV) [51,57]. 180° rotation shown in the right panel.

Biochemical analyses reveal that SaXPD binds G4 DNA with high specificity while exhibiting negligible affinity for duplex or single-stranded DNA. The catalytic core comprises two Rad51/RecA-like helicase domains (HD1 and HD2) spanned by the 4Fe–4S cluster and the Arch domains, and forms a substrate channel (Figure 4A,B). The 4Fe–4S cluster is indispensable for both structural integrity and catalytic function [53], and its redox sensitivity has been implicated in DNA-damage sensing. Crystal structures demonstrate that the 4Fe–4S cluster is a primary determinant of the enzymatic 5′→3′ translocation polarity and its capacity to recognize damaged DNA [54]. SaXPD-mediated G4 unwinding is strictly ATP-dependent [55]. To unwind substrates, the DNA must thread through the internal channel of XPD, whose dynamic opening and closing constitute the critical mechanistic feature of the helicase.

Compared with XPD, FANCJ possesses two additional structural modules, including a C-terminal domain that interacts with BRCA1 and an insertion element within the HD1 domain that harbors a nuclear localization signal (NLS), and an MLH1-binding site (Figure 4A). These features are absent in XPD and likely responsible for its inability to recognize or stably bind G4 structures [52,56]. Thus, FANCJ represents a new family member that has specifically evolved the capacity for G4 recognition and unwinding.

### 2.4. FANCJ Helicases

FANCJ is a canonical 5′→3′ helicase and the archetypal member of the XPD family with demonstrated G4 unwinding activity [58,59]. It efficiently resolves diverse G4 topologies and possesses a structurally defined G4-recognition site. The mutations of key lysine residues K141/K142 (AKKQ motif) within the HD1 domain abolished the binding of the G4 structure without compromising binding to or unwinding of single- or double-stranded DNA [52]. Unwinding is precise, reversible and repetitive: G4 binding is ATP-independent, whereas catalytic efficiency strictly requires ATP hydrolysis. This activity is functionally linked to DNA replication and damage tolerance [60]. FANCJ exhibits maximal catalytic activity as a dimer and prefers G4 substrates bearing a 5′ single-stranded overhang. Interestingly, RECQ1, another SF2 helicase, can also unwind G4 structures, indicating that G4 unwinding may be a universal property of the SF2 family [61]. Moreover, the G4-binding surface of FANCJ partially overlaps with its protein-interaction interface (e.g., the MLH1-binding site), suggesting that its activity can be modulated by protein partners [62,63]. For example, RPA (Replication Protein A) enhances FANCJ-mediated G4 unwinding approximately three-fold, whereas the G4 binding partner mismatch-repair factor MSH2–MSH6, competitively excludes FANCJ from the substrate and thereby impedes G4 metabolism.

FANCJ displays substrate-selectivity, it unwinds unimolecular G4s with the highest efficiency and exhibits lower activity toward tetramolecular G4s. FANCJ and the 3′→5′ helicase DHX36 can function synergistically, but their roles are distinct. DHX36 first generates single-stranded DNA downstream of the G4, facilitating CMG (Cdc45–MCM–GINS) bypass. Then FANCJ subsequently binds and resolves the G4 itself, enabling DNA polymerase progression [64].

### 2.5. RecQ Helicases

RecQ helicases constitute a conserved family of DNA unwinding enzymes that are indispensable for genome stability in both prokaryotes and eukaryotes. Among human RecQ paralogs, BLM, WRN and RECQ1 efficiently resolve G4 DNAs, and the helicase-and-RNaseD C-terminal (HRDC) domain is essential for this activity [65]. To date, high-resolution crystal structures capturing the G4-unwinding state have been reported only for human RECQ1 and CsRecQ (a RecQ homolog from *Cronobacter sakazakii*) [66]. Both structures decompose a single G tetramer through coordinated interactions. Current mechanistic insights are derived from low-resolution electron-microscopic reconstructions or mutagenesis–functional studies, representing an outstanding opportunity for homology modelling or cryo-EM.

**Figure 5:**
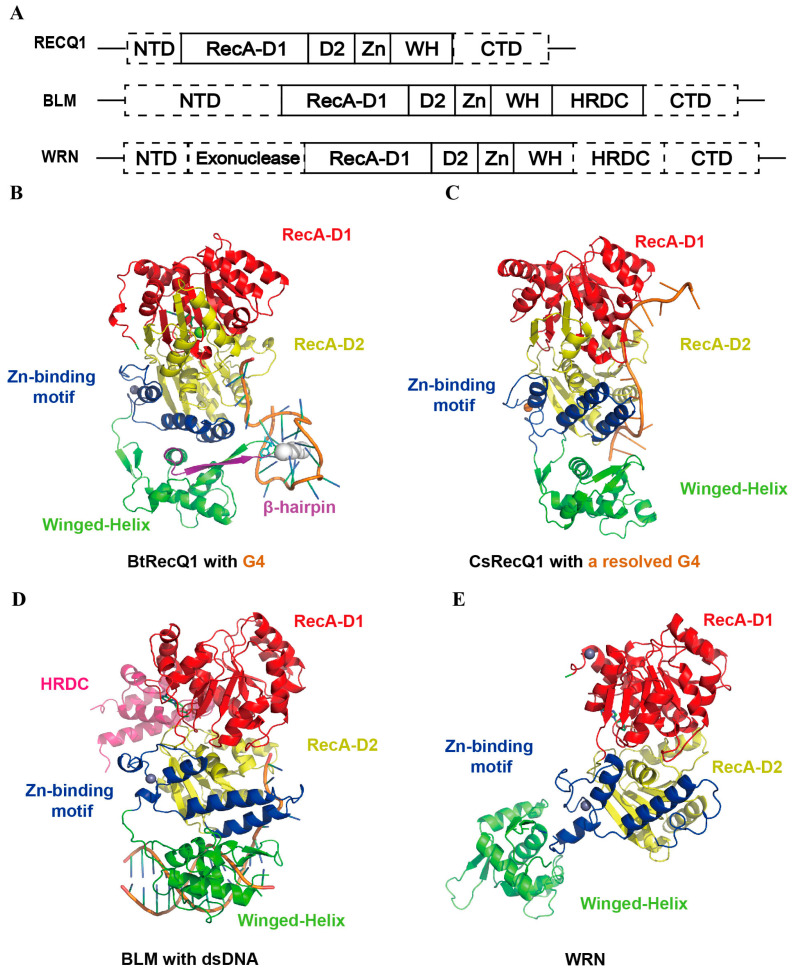
Structural schematic of RECQ1, BLM, and WRN. (**A**) The structural domains of RECQ1, BLM and WRN in the resolved crystal structure. The dash lines indicate the unresolved parts in the crystal structure. (**B**) Crystal structure of BtRecQ1 (PDB code: 9I1P) [61]. G4 DNA, orange; K^+^, white spheres. (**C**) Crystal structure of CsRecQ1 (PDB code: 6CRM) [66]. (**D**) Crystal structure of BLM (PDB code: 4CGZ) [68]. Zn^2+^, gray spheres. (**E**) Crystal structure of WRN (PDB code: 6YHR) [69].

RECQ1 oligomerizes upon G4 binding. Two G4 molecules associate via 5′-end-to-5′-end stacking to form a dimeric G4 substrate, which subsequently induces RECQ1 tetramerization. The D1, D2 and winged-helix (WH) domains of RECQ1 cooperatively recognize the G4 ribose–phosphate backbone and loop regions through hydrogen-bond and π-stacking interactions (Figure 5A) [61]. Unwinding is initiated by threading the 3′-single-stranded DNA (ssDNA) tail into the ssDNA-binding channel of RECQ1, thereby imposing a conformational rearrangement on the G4 tetrad and facilitating strand separation. A β-hairpin element interacts with tetrads, disrupting Hoogsteen hydrogen bonds (Figure 5B). As the ssDNA channel pulls the G4 3′-tail toward the D2 domain, the β-hairpin adopts a face-to-face orientation with the third G-tetrad (4G-8G-13G-17G) [61]. Driven by ATP hydrolysis, 3′→5′ ssDNA translocation advances the β-hairpin, whose apical aromatic/hydroxyl side chains (F561, T566 and Y564) wedge between the two tetrad layers and thereby disrupt the Hoogsteen hydrogen bonds. Consequently, the first G-tetrad disassembles into a G-triplex intermediate. After cleavage, the β-hairpin retracts with the translocating D2 domain, allowing G4 to restack or continue unfolding. RECQ1 repeats this cycle, achieving stepwise peeling rather than one-step unwinding. Then ATP-hydrolysis-driven translocation propels the β-hairpin through the G4 structure, progressively dismantling it. Comparative analysis reveals that human RECQ1 permits rapid post-unwinding G4 refolding, maintaining a dynamic equilibrium. In contrast, bovine RECQ1 engages the G4 tetrad through a tighter protein—DNA interface, resulting in less frequent refolding and more complete unwinding. In summary, the tetrameric assembly of RECQ1 provides a structural framework for understanding alternative lengthening of telomeres (ALT) and recurrent breast-cancer mutations, and the β-hairpin region emerges as a prospective anti-cancer drug target.

In the crystal structure of CsRecQ (*Cronobacter sakazakii* RecQ), the G4 is fully unwound (Figure 5C) [66]. The 3′-most guanine bases are fully unwound and sequestered within a guanine-specific pocket (GSP) unique to bacterial RecQ. The enzyme recognizes the G4/ssDNA junction, captures the extruded guanine within the GSP, thereby destabilizing the G4 scaffold, and translocates along the DNA to repeat the cycle. The liberated guanine can re-insert into the G4, giving rise to iterative unwinding—refolding cycles. However, the GSP is absent in eukaryotic RecQ homologs, indicating divergent prokaryotic and eukaryotic strategies for G4 processing. Mutations within the GSP afford a rational basis for species-selective inhibitor design [66].

BLM recognizes G4 structures via its RecQ C-terminal (RQC) domain, and the HRDC domain further enhances binding stability (Figure 5D). ATP binding elicits a conformational transition that allows BLM to handle complex DNA structures. Upon G4 recognition, the HRDC domain undergoes a further structural rearrangement that locks BLM onto the G4, enabling ATP-dependent translocation-coupled unwinding. BLM translocates in the 3′→5′ direction along the nucleic acid, unwinds the tetrad array from the 3′ side of the G4 in a stepwise manner [67]. Therefore, the HRDC domain is indispensable for BLM-mediated G4 resolution [68].

WRN (Werner syndrome helicase), a human RecQ family member, possesses 3′→5′ helicase and intrinsic 3′→5′ exonuclease activities that are essential for DNA repair, replication-fork restart, and telomere maintenance. WRN recognizes G4 structures through its winged-helix (WH) and zinc-binding (Zn) domains (Figure 5E) [69]. An insertion element within the D2 domain is proposed to destabilize the G4 scaffold. And ATP-hydrolysis-driven conformational changes between the D1 and D2 domains propel the single-stranded DNA through the helicase channel, thereby accomplishing G4 unfolding. Recent studies have revealed that microsatellite-instability-high (MSI-H) cancers exhibit a selective dependency on WRN, establishing the helicase as a validated anti-cancer target [70].

In terms of substrate specificity for unwinding G4 structures, DHX36 mainly unwinds parallel G4 structures, ScPif1 mainly unwinds antiparallel ones, while the remaining enzymes can unwind both types of topologies but with different efficiencies. DHX36 is the only type of helicase among the helicases discussed in this review that can unwind both DNA G4 and RNA G4 structures. The others are either specifically for DNA G4 or have extremely low RNA activity. Only the DHX36 unwinding direction is 3′→5′, while the others are 5′→3′. DHX36, with its DSM motif binding parallel G4 in a wide groove, efficiently unwinds parallel RNA or DNA-G4, with ≤20% residual activity against antiparallel G4s [44]. The Pif1 family shows a differentiation between eukaryotic and prokaryotic: the eukaryotic-derived ScPif1 forms a cation-π interaction through Arg324 in the wedge region with the 5′ end outermost G4, showing a significant preference for antiparallel or mixed-type G4, and the bacterial-derived ToPif1 uses the hydrophobic surface of the 1B-2B region to envelop the G4, with a binding rate not affected by topology and the unwinding rate mainly determined by the thermal stability of the G4. The XPD Arch/Fe-S cluster endows it with almost equal recognition capabilities for parallel and antiparallel G4, but strictly relies on 3′ single-strand tail loading and only acts on DNA, with an efficiency lower than FANCJ. While FANCJ strictly relies on the 5′ single-strand tail and the iron-sulfur cluster recognition mode, with a 2.5-fold higher unwinding rate for parallel G4 compared with antiparallel G4 [71]. RECQ1 also relies on the 3′ single-strand tail and prefers the parallel topology, but the rate of unwinding G4 is much lower than that of the homologous double strand, and it has almost no activity for RNA-G4. BLM and WRN retain 3′ single-strand tail dependence and a preference for parallel over antiparallel, but maintain 40–50% activity for RNA-G4, significantly different from the iron-sulfur cluster family. In summary, each helicase achieves precise division of labor for G4 structures and chemical diversity through three parameters: tail-loading direction, topology-recognition structure, and RNA tolerance.

## 3. The Influence of G4 Ligands on the Helicase Activity in Drug Design

Conventionally, transcription factors such as ATF4, MYC [72,73], and KRAS have been considered undruggable because they lack enzymatically active sites or present flat, extended surfaces. Targeting G4 structures in oncogene promoters has emerged as a promising strategy to address the challenges posed by undruggable and drug-resistant proteins such as MYC, BCL2, KRAS, and EGFR. G4 offers an alternative avenue for drug discovery: small molecules that stabilize G4 structures in the promoter regions of these genes can silence their transcription and thereby achieve indirect, yet selective, targeting [74,75]. Meanwhile, G4s represent promising targets for the development of new antifungal drugs with novel mechanisms [76]. Current G4-directed drug design can be broadly categorized into three strategies: stabilization, destabilization, and multi-target synergy. The predominant approach employs small-molecule ligands that stabilize G4 structures, thereby impeding helicase-mediated unfolding and obstructing replication or transcription machineries, ultimately provoking DNA damage or gene silencing. In addition, pharmacological acceleration of G4 unwinding can prevent replication-fork stalling and reduce genomic instability. Multi-target synergy entails concurrent modulation of G4 structures and their upstream or downstream regulatory proteins, or of the surrounding chromatin environment, to enhance therapeutic efficacy while minimizing off-target toxicity. For example, the first telomerase inhibitor Imetelstat was approved in 2024 for medical use by the FDA [77].

A growing body of evidence demonstrates that small-molecule–mediated stabilization of oncogenic G4 structures can down-regulate the transcription of driver genes and thereby retard tumor progression. These ligands have been shown to antagonize multiple helicases in a structure-selective manner. For Pif1, Phen-DC3 markedly impedes unwinding velocity through robust G4 stabilization, whereas the G4 ligand BRACO19 exerts negligible influence on Pif1 rate [78,79]. TrisQ and related scaffolds achieve >50% inhibition of Pif1-catalyzed resolution of c-MYC or c-KIT G4 substrates, reducing reaction velocity by more than twofold without detectable activity against single- or double-stranded DNA, confirming that the blockade is strictly G4-dependent [80].

For DHX36, the G4-stabilizing ligands PDS and Phen-DC3 decrease its unwinding velocity by 70- to 100-fold without compromising either ATP hydrolysis or substrate binding [81,82]. Telomestatin (TMS) similarly impedes the progression of both DHX36- and FANCJ-mediated G4 unwinding [83].

Regarding FANCJ, Phen-DC3 and Phen-DC6 exhibit nanomolar potency that is strictly topology-dependent, reducing unwinding rates by several hundred- to thousand-fold [59]. TMS essentially abolishes FANCJ activity at nanomolar concentrations, driving the reaction rate to background levels [60]. The anti-cancer drug RHPS4 stabilizes G4 structures, making them difficult to unwind. This, in turn, increases the demand for the unwinding activity of BLM/FANCJ. Targeting FANCJ can enhance the efficacy of G4-stabilizing anti-cancer drugs (such as RHPS4), providing a theoretical basis for combined treatment [84].

For BLM, three small-molecule ligands—L1H1-7OTD, PDS and Phen-DC3—markedly slow the unwinding of human telomeric G-quadruplex (hGQ), decreasing the unwinding frequency to approximately 1/2~1/3 of the control value [85]. A trisubstituted acridine operating at sub-micromolar concentrations attenuates BLM-catalysed G4 resolution by >50%, an effect attributed to impaired DNA binding [86].

With respect to WRN, NSC 19630 specifically restricts the motor domain, reducing the helicase velocity on forked DNA substrates without significantly affecting its ATPase or exonuclease activities [87]. Additional sub-micromolar compounds likewise diminish WRN unwinding rates [86].

The natural alkaloids berberine and coptisine markedly stabilize the G4 formed within the KRAS promoter, thereby stalling DNA polymerase progression and provoking replication arrest. Although helicases such as BLM or WRN were not directly assayed, excessive stabilization of the G4 motif is expected to obstruct their translocation, allowing the inference that these ligands indirectly suppress G4 unwinding [88].

Notably, divergent increases in thermal stability (ΔTm) among ligands do not necessarily translate into different inhibitory outcomes against BLM, underscoring that thermostabilization per se is an unreliable predictor of helicase blockade, direct interference with the kinetics of unwinding is the decisive parameter [85].

At the cellular level, PDSI impedes WRN by reinforcing G4 structures, diminishing its DNA association, attenuating unwinding velocity, and selectively curtailing proliferation of BRCA2-deficient cells [89,90]. Mechanistically, TMS and related ligands “physically lock” G4 motifs through π–π stacking, rendering them refractory to FANCJ-mediated resolution. FANCJ depletion further amplifies TMS-induced DNA damage by prolonging G4 persistence and replicative stress. Analogous modes of action—evidenced for OTDs, CX-5461, and other G4 stabilizers—consistently rely on helicase inhibition to exert their anticancer effects [83].

## 4. Challenges of Reading Through G4s in Nanopore Sequencing

Nanopore sequencing, a third-generation sequencing technology, offers several advantages over earlier sequencing technologies [91], including high sequencing speed, ultra-long read lengths, simultaneous base modification detection (e.g., methylation), and single-molecule real-time analysis with convenience and cost-effectiveness. These features have enabled its widespread application in diagnosis, treatment, and scientific research. Furthermore, helicases are employed as molecular motors in nanopore sequencing to unwind double-stranded DNA (dsDNA) at a specific rate, and the current changes induced by helicases can be measured with higher accuracy, prolonged and stable current signals for each nucleotide [92,93,94,95]. Subsequently, the unwound single strands are translocated through biological nanopores, with current shifts recorded in real time.

To date, several helicases have been utilized as motors, including the superfamily 2 (SF2) helicase Hel308 [96], the mutated Dda [97], and Tra. Nucleic acids without specialized secondary structures typically translocate through the channel continuously; however, the stability of G4 DNA poses a significant challenge. G4 structures act as physical barriers, impeding the smooth translocation of DNA molecules through nanopores and leading to signal interruptions (due to pore blockage) or sequencing errors. To overcome this translocation obstacle, electrophoretic force can be applied to pull and unwind nucleic acids, but this process is often excessively prolonged (>4 min) [98] and not always conducive to signal stability. More specifically, the “barrier effect” of G4s in nanopore systems can manifest as physical blockage/conformational trapping and perturbations to motor kinetics.

In practical sequencing workflows, Oxford Nanopore Technologies (ONT) and related platforms rely on motor enzymes to feed single-stranded nucleic acids into the pore with near-constant stepping, if stable G4s or G4-interconverting intermediates form on the template, pronounced pausing, back-stepping, or “skipping” may occur, thereby reducing throughput and contributing to systematic errors. Consistent with this, a systematic analysis across sequencing technologies suggests that, at motifs capable of forming non-B DNA structures (including G4), ONT data show detectable local shifts in sequencing success rates and error profiles, and deletion errors at G4 motifs tend to be elevated [99].

Moreover, the size-selective properties of nanopores do not allow larger conformations, including the propeller fold of G4s, to enter the opening of the vestibule, often requiring propeller G4s to unfold outside of the protein nanocavity. Overall, G4s pass through the nanopore slowly and discontinuously. Therefore, solutions should not rely solely on increasing electrophoretic force (which may be slow and unfavorable for signal stability [98]), but rather require a more “active” structural resolution strategy—namely, introducing ATP-dependent G4-unfolding helicase modules so that the “unfolding—translocation—readout” process remains controllable at the single-molecule scale [92,100].

Based on this, G4 helicases have been incorporated into nanopore sequencing platforms. G4 helicases, functioning as a class of molecular motors, harness the energy released by ATP hydrolysis to disrupt the Hoogsteen hydrogen bonds within G4 structures, restoring them to single- or double-stranded conformations and thereby providing a more unobstructed template for replication, transcription, or sequencing. Introducing G4 helicases into sequencing systems offers multiple advantages. First, G4 structures can be effectively unwound, ensuring strand continuity and promoting smoother movement of molecules through the nanopore. For instance, single-molecule nanopore monitoring techniques have been employed to observe the unwinding process of DNA/RNA G4 structures in real time by helicases SARS-CoV nsp13 or RTEL1 [100]. Second, unwinding G4 structures typically yields clearer and more accurate electrical signals, thereby improving the accuracy and resolution of sequencing [95,97,101,102,103]. Third, by more effectively managing complex secondary structures such as G4, nanopore sequencing may better address “difficult” genomic regions, including G4-rich telomeric regions and specific viral genomes. Recent advances, such as ONT’s R10, have intensified the demand for higher-resolution data-processing algorithms. In this context, integrating G4 helicases, such as XPD [104], may enable more precise control over both the translocation kinetics and conformational landscape of nucleic-acid substrates, yielding cleaner ionic-current signatures and thereby enhancing sequencing resolution [102].

Overall, using helicases with defined G4 recognition and unfolding pathways as “replaceable motors” may reduce the risk that G4 structures disrupt sequencing continuity through ATP-driven, directional unfolding. Nanopore-based monitoring systems can be used to screen selectivity and rates across different topologies and RNA/DNA G4 substrates quantitatively [100,105]. Meanwhile, it should be noted that nanopore sequencing often benefits from high-salt conditions to improve SNR, whereas commonly used motor enzymes may exhibit limited activity under such conditions. Recent studies on helicases from acidophilic organisms and their engineering to improve high-salt tolerance and translocation capability provide promising directions for developing “next-generation motors” better adapted to nanopore conditions; however, whether they can reliably read through G4 in real sequencing workflows still requires further rigorous validation [106,107].

## 5. Conclusions and Perspectives

A substantial body of evidence demonstrates that G4s function as epigenetic and regulatory elements in a variety of aberrant biological processes, participating in DNA replication, transcription, and translation. G4 formation can suppress DNA methylation and modulate nucleosome assembly, thereby serving as distinctive epigenetic signatures. Moreover, the presence of G4s may induce site-specific mutagenesis, gene deletion–junction events, transpositions, rearrangements, and copy-number alterations, all of which constitute major sources of genomic instability and disease pathogenesis [16,108,109]. Structural characterization of G4–ligand complexes provides critical insights into molecular recognition mechanisms of ligands and facilitates the rational design of novel therapeutics [110]. Based on the determined complex structures, it is evident that natural small molecules achieve specific recognition of G4s through a combination of interactions, including π–π stacking, hydrogen bonding, electrostatic interactions, and steric effects.

Despite the growing enthusiasm for G4-targeted therapeutics, several bottlenecks persist that mirror those encountered in other nucleic-acid-directed programs. First, achieving nucleotide-level selectivity remains arduous: cationic or π-rich ligands often recognize canonical duplex DNA or alternative non-B conformations with comparable affinity, increasing the risk of off-target transcriptional or replicative stress [111]. Second, the lifetime of a given G4 in vivo is governed by its genomic context, local superhelical density, epigenetic states, and the competing action of helicases [10]. For example, G4 stability is highly sensitive to methylation of cytosines or i-motifs (quadruplex cytosines from the G4 complementary sequence) in loops and may affect the binding of ligands [8,112]. Consequently, a ligand must retain high on-rate and slow off-rate under nucleotide- and protein-concentrated conditions that cannot be fully recapitulated in vitro. Third, efficient nuclear delivery is non-trivial—positively charged scaffolds that favour G4 interaction frequently become trapped in endosomes or bound to plasma proteins, limiting the free fraction that reaches chromatin. Finally, the ubiquity of G4 motifs across promoters, telomeres, and RNA 5′-UTRs obliges rigorous toxicological profiling to exclude global perturbation of housekeeping genes or exhaustion of hematopoietic stem cells.

To confront these challenges, the field is coalescing around an integrated discovery pipeline. Structure-guided design cycles now routinely exploit co-crystal structures (X-ray) or solution ensembles (NMR) of G4–ligand complexes to introduce shape- and electrostatic-matched heteroatoms that disfavour duplex insertion. Parallel high-throughput screening of natural-product libraries—enriched in alkaloids and flavonoids—continues to supply chemotypes with unprecedented selectivity indices that can be subsequently optimized by medicinal chemistry. Selectivity and functional engagement are quantified at the genome-scale: ChIP-seq maps ligand-induced G4 stabilization sites, G4-seq delineates the native fold landscape, and RNA-seq flags both intended oncogene repression and inadvertent transcriptome dysregulation. Promising candidates are then advanced into murine patient-derived xenograft (PDX) and humanized mouse models in which pharmacokinetics, maximum tolerated dose, and on-mechanism toxicities (e.g., myelosuppression, hepatotoxicity) are benchmarked against genetic knockdown of the corresponding G4-associated oncogene. Collectively, this iterative feedback loop is steadily converting G4 biology from an academic curiosity into a druggable axis of cancer therapy.

In the context of nanopore sequencing, it is particularly promising to translate the “structural basis” of G4 helicase recognition and unfolding discussed in this review into a synergistic strategy that combines motor screening/engineering with signal modeling. Without overstating current applicability, G4 helicases with controllable stepping and selective unfolding capability may serve as enhancer elements for hard-to-read segments, improving read-through performance and result reliability in G4-enriched regions [100,107,113]. Although G4 helicases hold broad application prospects in nanopore sequencing, several challenges remain, such as enzymatic specificity and efficiency, integration and control, and data analysis. In summary, as nanopore sequencing technology matures and G4 helicase research deepens, the application of G4 helicases in nanopore sequencing is poised to bring revolutionary changes to genomics research, disease diagnosis, and drug development, particularly in the precise identification and analysis of complex genomic structures.

## Data Availability

No new data were created or analyzed in this study. Data sharing is not applicable to this article.

## Abbreviations

The following abbreviations are used in this manuscript:

ALT	Alternative lengthening of telomeres
CMG	Cdc45–MCM–GINS
CsRecQ	*Cronobacter sakazakii* RecQ
dsDNA	Double-stranded DNA
DSM	DHX36-specific motif
G4	G-quadruplex
GRS	G4- recognizing surface
GSP	Guanine-specific pocket
HD	Helicase domain
hGQ	Human telomeric G-quadruplex
HRDC	Helicase-and-rnased C-terminal
MSI-H	Microsatellite-instability-high
NER	Nucleotide-excision repair
NLS	Nuclear localization signal
OB	Oligonucleotide/oligosaccharide-binding
ONT	Oxford Nanopore Technologies
PDX	Patient-derived xenograft
RPA	Replication protein a
RQC	RecQ C-terminal
SaXPD	*Sulfolobus acidocaldiarius* XPD
SF1	Superfamily 1
SF2	Superfamily 2
ssDNA	Single-stranded DNA
TaXPD	*Thermoplasma acidophilum* XPD
TMS	Telomestatin
ToPIF1	*Thermus oshimai* Pif1
UTRs	Untranslated regions
WH	Winged helix
WRN	Werner syndrome helicase
